# Identification of placenta-specific protein 1 (PLAC-1) expression on human PC-3 cell line-derived prostate cancer stem cells compared to the tumor parental cells

**DOI:** 10.1007/s12672-024-01121-x

**Published:** 2024-06-28

**Authors:** Pooya Farhangnia, Roya Ghods, Reza Falak, Amir-Hassan Zarnani, Ali-Akbar Delbandi

**Affiliations:** 1 Department of Immunology, School of Medicine, Iran University of Medical Sciences, Tehran, Iran; 2 Immunology Research Center, Institute of Immunology and Infectious Diseases, Iran University of Medical Sciences, Tehran, Iran; 3 Oncopathology Research Center, Iran University of Medical Sciences, Tehran, Iran; 4 Department of Molecular Medicine, Faculty of Advanced Technologies in Medicine, Iran University of Medical Sciences, Tehran, Iran; 5 Department of Immunology, School of Public Health, Tehran University of Medical Sciences, Tehran, Iran; 6 Reproductive Sciences and Technology Research Center, Department of Immunology, School of Medicine, Iran University of Medical Sciences, Tehran, Iran

**Keywords:** PLAC-1, Cancer stem cell, Placenta-specific protein 1, Immunotherapy, CD44, CD133

## Abstract

Placenta-specific protein 1 (PLAC-1) is a gene primarily expressed in the placenta and the testis. Interestingly, it is also found to be expressed in many solid tumors, and it is involved in malignant cell features. However, no evidence has been reported regarding the relationship between PLAC-1 and cancer stem cells (CSCs). In the current research, we explored the expression of the PLAC-1 molecule in prostate cancer stem cells (PCSCs) derived from the human PC-3 cell line. The enrichment of PCSCs was achieved using a three-dimensional cell culture technique known as the sphere-formation assay. To confirm the identity of PCSCs, we examined the expression of genes associated with stemness and pluripotency, such as SOX2, OCT4, Nanog, C-Myc, and KLF-4, as well as stem cell differentiation molecules like CD44 and CD133. These evaluations were conducted in both the PCSCs and the original tumor cells (parental cells) using real-time PCR and flow cytometry. Subsequently, we assessed the expression of the PLAC-1 molecule in both enriched cells and parental tumor cells at the gene and protein levels using the same techniques. The tumor cells from the PC-3 cell line formed spheroids with CSC characteristics in a non-adherent medium. The expression of SOX2, OCT4, Nanog, and C-Myc genes (*p* < 0.01), and the molecules CD44 and CD133 (*p* < 0.05) were significantly elevated in PCSCs compared to the parental cells. The expression of the PLAC-1 molecule in PCSCs showed a significant increase compared to the parental cells at both gene (*p* < 0.01) and protein (*p* < 0.001) levels. In conclusion, it was indicated for the first time that PLAC-1 is up-regulated in PCSCs derived from human PC-3 cell line. This study may propose PLAC-1 as a potential target in targeted therapies, which should be confirmed through further studies.

## Introduction

Prostate cancer (PC) is the second most common cancer and the fifth major cause of cancer mortality among men in 2020, with an expected 1.4 million new cases and 375,000 deaths worldwide [1]. There are some anticancer armamentariums against PC, including surgery, chemotherapy, androgen-targeted drugs, radionuclides, poly(ADP-ribose) inhibitor olaparib, and immunotherapies approaches such as PROSTVAC and Sipuleucel-T [2]. However, PC may remain latent for a significant amount of time after therapy [3], accounting for tumor recurrence.

There is overwhelming evidence to support that prostate cancer stem cells (PCSCs) play a crucial role in the PC progression [4–6]. Prostate stem cells (PSCs) are found in the basal and luminal layers of the prostate and are the targets of oncogenic transformation, suggesting that PCSCs play a role in the onset of PC [5]. According to the cancer stem cell (CSC) theory, a subset of cancerous cells with stem cell characteristics, especially self-renewal ability, generation of other highly differentiated cell types, are at the top of the tumor cell hierarchy and are a key factor in the development of tumors [7]. CSCs have been associated with conventional therapies resistance and poor prognosis to therapy, Epithelial-mesenchymal transition (EMT), metastasis, tumor recurrence, tumor immune evasion, and in human cancers [7–9].

Over the last two decades, immunotherapy has been a revolutionary new pillar in cancer treatment. However, a major hurdle in cancer immunotherapy is identifying appropriate tumor-specific antigens to make targeted therapy achievable with fewer normal cells being impaired [10]. In this regard, placenta-specific protein 1 (PLAC-1) is an attractive candidate for targeted immunotherapeutic approaches [11]. PLAC-1 molecule has been introduced as a bridge between placentation and tumor development and growth [10]. Originally, Cocchia et al. [12] introduced the PLAC-1 gene as one of the oncoplacental genes, which encodes a small protein containing 212 amino acids. Substantially, PLAC-1 molecule is expressed by trophoblast cell lineage, and it has a fundamental role in placental function and development [13–17].

First, Koslowski et al. [11] demonstrated that PLAC-1 is involved in malignant cell processes, and it is ectopically activated in many human malignancies. Furthermore, PLAC-1 localization was indicated on the surface of tumor cells, making it accessible for antagonistic monoclonal antibodies [11]. PLAC-1 also has no discernible expression in any other healthy human tissue [11]. After that, numerous studies have addressed the association between PLAC-1 and cancer, which indicated the activation and expression of this molecule in a wide variety of liquid and solid tumors, including acute leukemia [18], melanoma [19], breast [11, 20–25], lung [11, 20, 21, 26, 27], nasopharynx [28], liver [20, 26, 29], colon [20, 21, 26, 30–36], stomach [11, 37–39], prostate [40, 41], ovary [11, 42–44], uterus [45, 46], cervix [20, 47, 48], head and neck [49], and pancreas [50]. There are numerous empirical studies indicating the role of PLAC-1 in tumor cell proliferation, migration, and invasion [10]. A mouse model of melanoma showed that immunizing with PLAC-1 triggers robust anti-tumor responses and increases survival duration [51]. However, no previous study has investigated the expression of PLAC-1 in CSCs.

In 2009, a list of 75 cancer antigens was proposed by the National Cancer Institute (NCI) based on several criteria, including therapeutic function, immunogenicity, the role of the antigen in oncogenicity, specificity, expression level and percent of antigen-positive cells, stem cell expression, number of patients with antigen-positive cancers, number of antigenic epitopes, and cellular location of antigen expression [52]. In this ranking, PLAC-1 molecule was ranked 47th [52].

It has been revealed that PLAC-1 expression was up-regulated in a stepwise manner from benign prostatic hyperplasia to PC, which had the greatest levels of this molecule. Indeed, PLAC-1 expression is increased in accordance with increasing grade and Gleason score of PC [40]. Additionally, there is an association between CSC and increased severity of malignancy in PC [5]. Thus, this study aimed to address the following research question: Is PLAC-1 expression higher in the PCSCs phenotype-enriched population than in their parental PC cell line?

## Materials and methods

### Study design

**Fig. 1:**
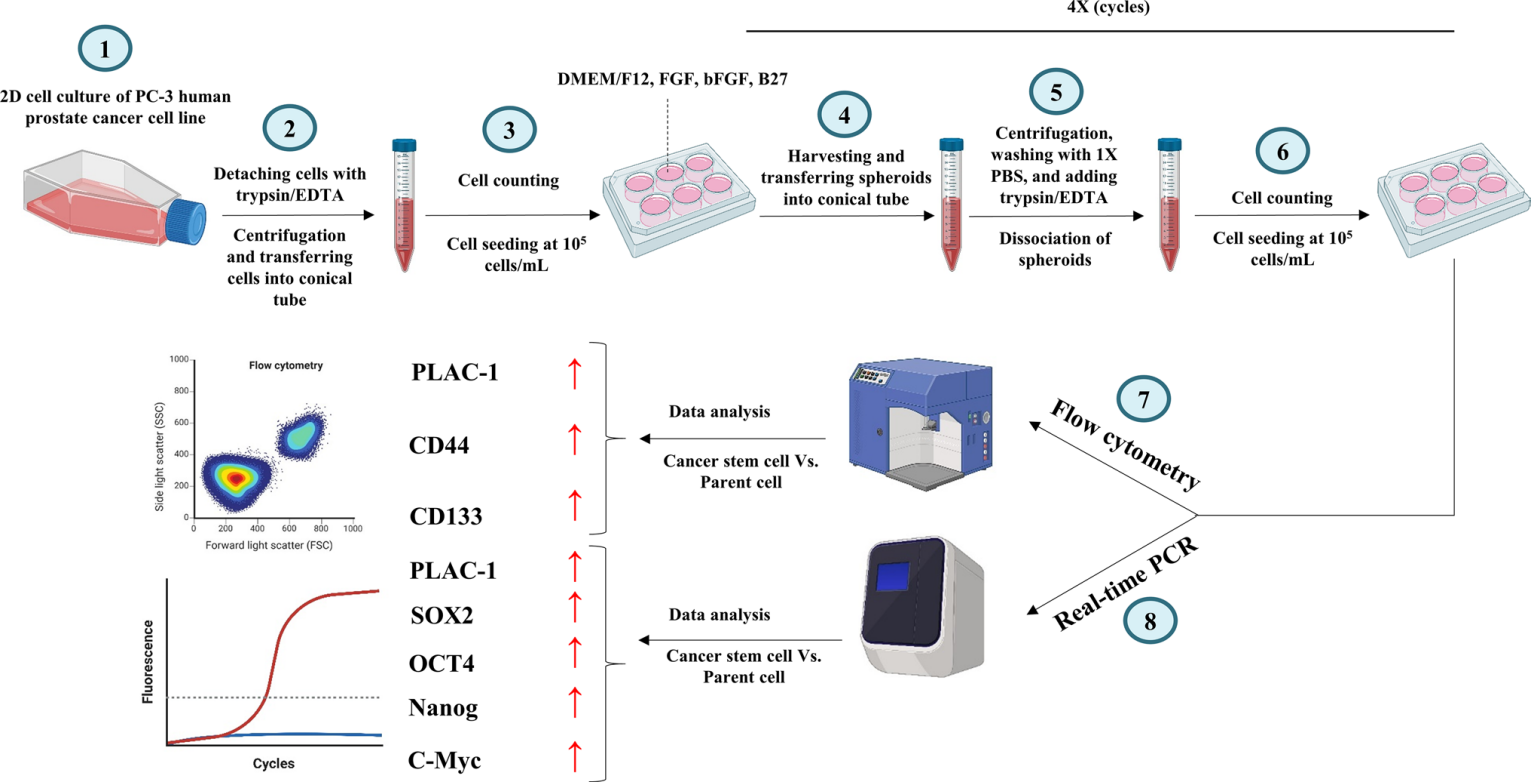
Experimental scheme of the study

Initially, a human prostate carcinoma cell line (PC-3) was cultured and subsequently seeded in ultra-low attachment plates following cell counting. The culture medium was enriched with specific growth factors over several days, leading to the formation of CSC colonies. These colonies were harvested, dissociated, and re-seeded in ultra-low attachment plates. This process was repeated up to four iterations. Subsequently, the expression of PLAC-1 and CSC-related markers was assessed utilizing flow cytometry and real-time PCR methodologies (Fig. 1). The procedural steps are delineated in the subsequent sections.

### Cell lines and culture conditions

The PC-3 human prostate adenocarcinoma cell line was obtained from the Avicenna Research Institute (ACECR, Tehran, Iran) and was tested for spheroid formation. Cells were cultured in Dulbecco’s Modified Eagle Medium/Nutrient Mixture F-12 (DMEM/F-12, Gibco, Germany) supplemented with 10% fetal bovine serum (Placenta-specific molecule 1, Gibco, Germany), 100 U/mL penicillin, 100 µg/mL streptomycin antibiotics (Gibco, Germany), and 2 mM l-glutamine (Gibco, Germany). Cultures were maintained under standard cell culture conditions at 37 ℃, 5% CO_2_, and 95% humidified incubator and passaged in 70–90% confluence, as previously described by Gheytanchi and colleagues [53].

### Preparation of poly‑HEMA coated cell culture dishes

In order to thoroughly dissolve the polymers, 1.2 g of poly-HEMA (Sigma, USA) was rotated overnight in 100 mL of 96% ethanol to form the 1.2% poly-HEMA solution. After being centrifuged at 400 × *g* for 30 min to remove any remaining particles, 0.22 µm filters were used to filter the solution. The tissue culture dishes were coated with poly-HEMA solution (1.2 mL per each well of six well plate or 2.5 mL per T25 tissue culture flask) under a biosafety laminar flow hood at room temperature an overnight to evaporate ethanol completely. Finally, the plates were washed with phosphate buffered saline (PBS) and stored at 37 ℃ incubator for future use, as previously reported by Gheytanchi and colleagues [53].

### Spheroids culture

The procedure for cultivating spheroids was performed following a previously described protocol [53]. PC-3 spheroids were generated by free-floating spheroid method on poly-HEMA-coated plates. The cells were detached using 0.05% trypsin/EDTA (Gibco, Germany). After trypsin inactivation, the single cells were washed twice with phosphate-buffered saline (PBS) and pre-warmed serum-free medium. Then, single-cell suspensions were seeded into poly-HEMA coated dishes (10^5^ cells/mL) per 2 mL of serum-free medium (DMEM/F12, Gibco, Germany), which was supplemented with 20 ng/mL epidermal growth factor (EGF; Royan Institute, Tehran, Iran), 10 ng/mL of basic fibroblast growth factor (bFGF; Royan Institute, Tehran, Iran), 2% B27 supplement (Gibco, Germany), 2 mM L-glutamine and 1% penicillin–streptomycin. Cultures were cultivated for up to 14 days. The culture medium was gently supplemented with additional 2% B27, bFGF and EGF every 3 days.

### Secondary sphere-formation assay

Spheroids were collected and physically and enzymatically separated using trypsin/EDTA and gentle pipetting in order to study the capacity of prostaspheres to generate new spheroid generations. After counting, the single cells were re-plated in serum-free spheroid media at the same densities and culture conditions as described above for three successive passages (P1-P4).

### Quantitative real‑time PCR analysis

Real-time PCR was used to analyze the gene expression. These genes included PLAC-1 and stemness genes such as KLF4, OCT4, SOX2, NANOG, and C-MYC. Spheroids were harvested a day before structural disintegration (day 9 for PC-3 prostaspheres). The total RNA was then extracted from parental and spheroid cells TRIzol Reagent (Sinaclon, Tehran, Iran) according to the manufacturer’s instructions. After measurement of RNA quantity and quality by Nanodrop (ThermoFisher Scientific, USA), cDNA was synthesized with 1 μg of total RNA using an Easy cDNA Synthesis Kit (Parstous, Iran) [53]. Real-time polymerase chain reaction (RT-PCR) was performed using BioFACT^™^ 2X Real-Time PCR Master Mix (For SYBR^®^ Green I, BioFACT, South Korea) on the Rotor-Gene Q Light Cycler (Qiagene, Germany) with the following conditions: 45 two-step amplification cycles of 95 ℃ for 15 s and 64 ℃ for 30 s. As the internal reference gene, glyceraldehyde-3-phosphate dehydrogenase (GAPDH) was used to quantify the relative expression levels of the target genes by using the 2^−ΔΔCT^ method. RT-PCR primers are listed in Table 1.

**Table 1:** Primers used for quantitative RT-PCR

Reverse primer	Forward primer	Gene
5′-TGGTGAAGACGCCAGTGGA-3′	5′-GCACCGTCAAGGCTGAGAAC-3′	GAPDH
5′-CCATGAACCAGTCTATGGAG-3′	5′- CACCAGTGAGCACAAAGCCACATT-3′	PLAC-1
5′-TCTGCGAGCTGGTCATGGAGTT-3′	5′-GCTACAGCATGATGCAGGACCA-3′	SOX2
5′- AAAGCGGCAGATGGTCGTTTGG-3′	5′- CCTGAAGCAGAAGAGGATCACC-3′	OCT4
5′-CGTCACACCATTGCTATTCTTCG-3′	5′-CTCCAACATCCTGAACCTCAGC-3′	Nanog
5′-TCGGTCGCATTTTTGGCACTGG-3′	5′-CATCTCAAGGCACACCTGCGAA-3′	KLF-4
5′-CAGACTCTGACCTTTTGCCAGG-3′	5′-CCTGGTGCTCCATGAGGAGAC-3′	C-Myc

### Flow cytometry

The percentage of PC-3 spheroid cells that exhibit CSC markers relative to parental cells was measured using flow cytometry. The parental and spheroid cells were dissociated with trypsin/EDTA and were washed with PBS twice. The dissociated cells were counted using Trypan blue exclusion assay [53], and if cell viability was more than 90%, they were evaluated for PLAC-1 and CSC markers expression. The following antibodies were used: anti-PLAC-1 (1:100, gifted from ACECR, Tehran, Iran), anti-CD44 (1:30, BD, USA), anti-CD133 (1:300, BD, USA). All antibodies were incubated with 3 × 10^5^ cells for 30 min at 4 ℃. Sheep anti-mouse IgG-FITC (1:100) (ACECR, Tehran, Iran) was used as secondary antibody. The percentage of PLAC-1 positive and CSC marker positive cells were evaluated using a BD FACSCalibur^™^ Flow Cytometer and FlowJo VX software was used to analyze the data.

### Statistical analysis

Data were reported as the mean ± standard deviation for each group. Mann–Whitney statistical test was performed for analyzing corresponding genes and proteins between two groups. GraphPad Prism version 8.0 (GraphPad Software, La Jolla, CA, USA, www.graphpad.com) for Windows was used to examine the differences between the control (parental) and spheroid groups. A p-value < 0.05 was accepted as a statistically significant difference between groups.

In this study, 2D and 3D cell culture experiments were conducted in four independent runs. Concerning the expression of fundamental genes, the experiments were carried out in two independent runs, with each run being performed in triplicate. PLAC-1 gene expression was assessed in three independent experiments, with each experiment being conducted in duplicate. For flow cytometry experiments, investigations into the expression of CD44 and CD133 molecules were undertaken in two independent runs, with each run being repeated twice. The expression of the PLAC-1 molecule was analyzed by flow cytometry in four independent runs, each run duplicated.

## Results

### PC-3 cell line generates three-dimensional (3D) spheroids

**Fig. 2:**
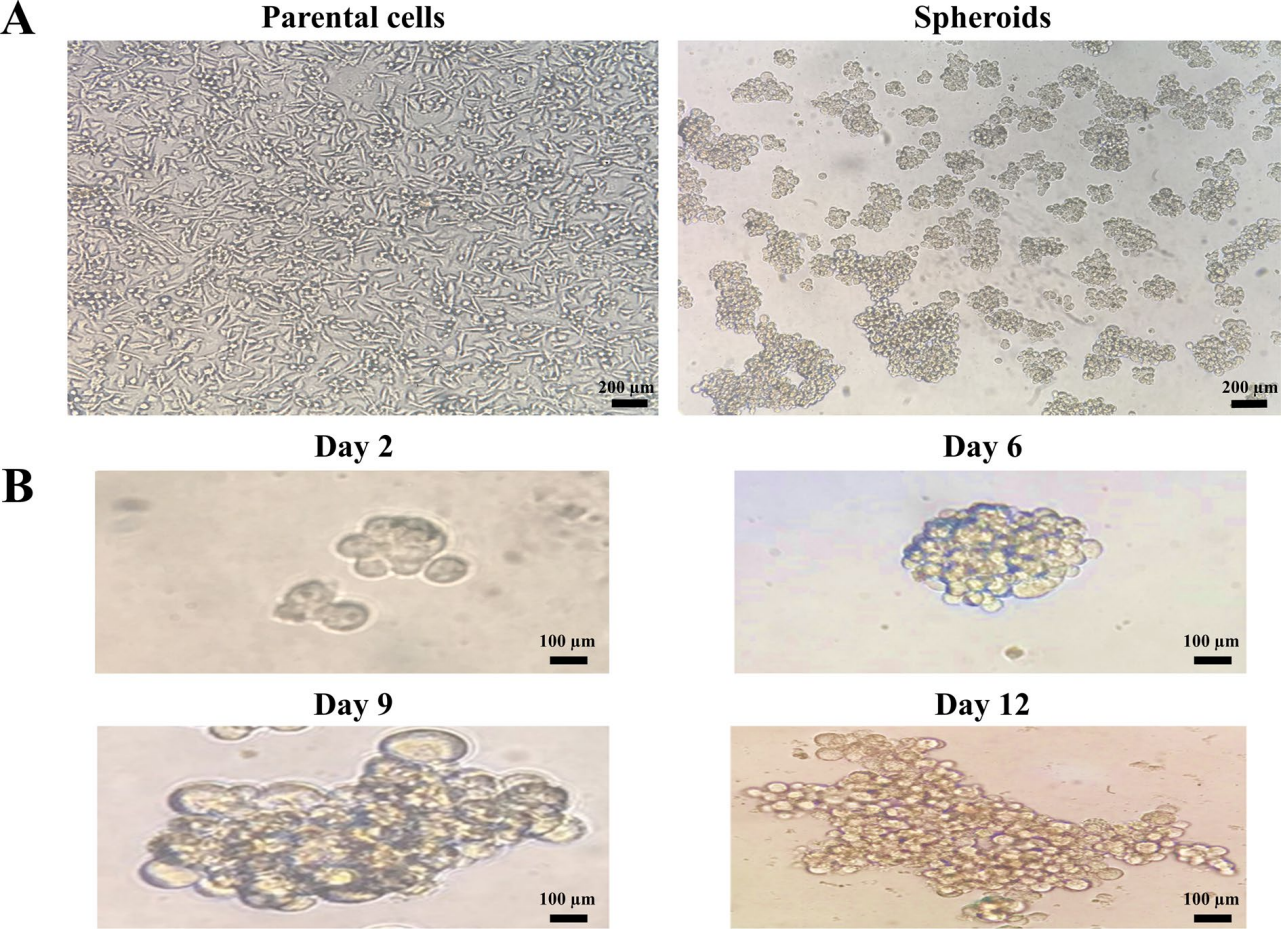
The formation of spheres composed of cancer stem cells derived from PC-3 cell line. **A** PC-3 parental cells (2D cell culture) vs spheroid colonies (3D cell culture). **B** The formation of prostaspheres on days 2, 6, 9 and 12 is shown. Spheres derived from PC-3 cell line form large organizations with relatively irregular edges

First, we tested whether the PC-3 cell line could generate 3D spheroids. Spheroid formation in non-adherent conditions on poly-HEMA coated dishes at different cell densities were applied to generate of spheroids from PC-3 adherent cells. As observed from Fig. 2A, PC-3 cell line generated discernable 3D spheroids in serum-free media using free-floating culture in non-adherent conditions on poly-Hema. PC-3-derived spheroids appeared large and loose in terms of organization after 72-h incubation and became more compact and dense during 12 days of culture (Fig. 2B).

### Enrichment of CSC sub-populations in formed spheroids

We used three different approaches to examine PCSC enrichment in generated spheroids in comparison to adherent counterparts: (1) secondary SFA whereby stemness aspects of cancer cells are maintained by using PCSCs capacity for self-renewal; (2) evaluation of the expression of significant stemness genes, including as SOX2, C-MYC, OCT4, KLF4, and NANOG, which act as master regulators of pluripotency and self-renewal in CSCs; and (3) quantifying the expression of potential PCSCs surface markers such as CD44 and CD133 by flow cytometry.

**Fig. 3:**
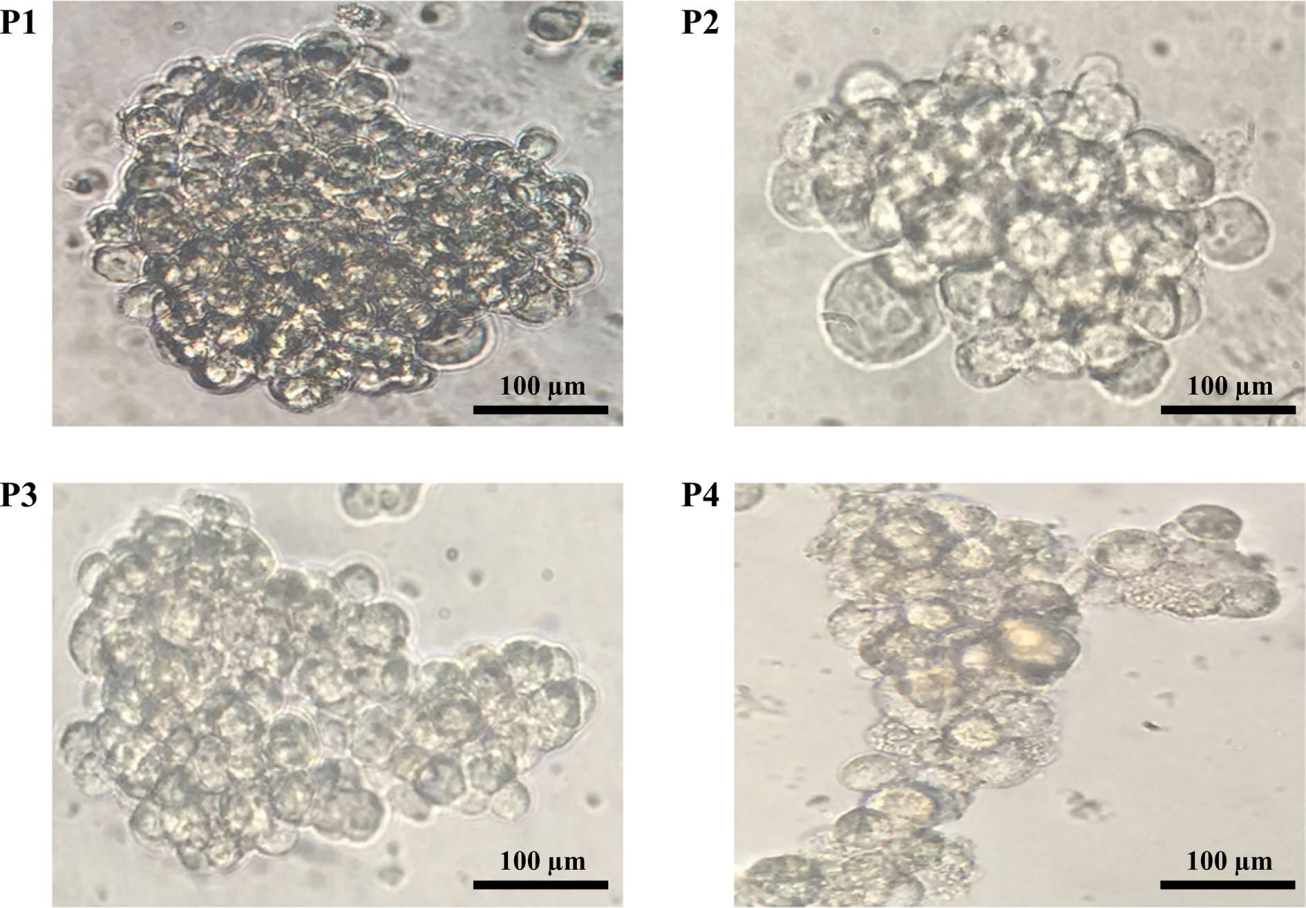
Serial spheroid formation capacity of prostaspheres. To assess the self-renewal features of the generated spheroids, secondary spheroid formation was conducted by serial passaging. Primary spheres derived from PC-3 produced spheroid colonies for four consequence passages (P1-P4)

**Fig. 4:**
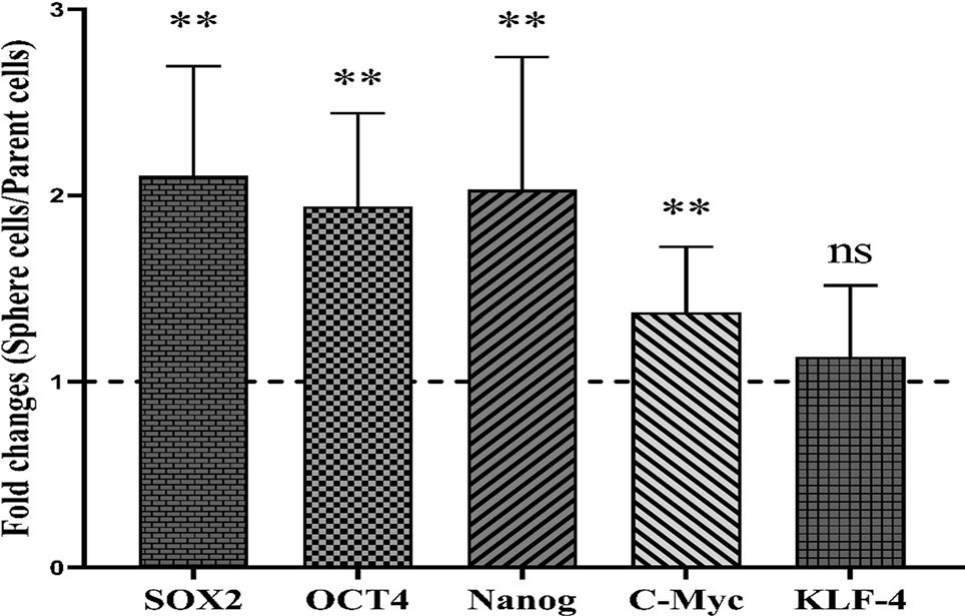
Increased expression of stemness regulator genes in PC-3-derived spheroids compared to adherent counterparts. The quantitative real-time PCR analysis of PC-3 spheroids revealed the high expression of key stemness genes OCT4, SOX2, NANOG, and C-Myc compared to PC-3 parental cells with the highest expression level of SOX2. Data are presented as mean ± SD from two independent experiments. The fold change of genes in cell spheroids was investigated after 14 days of culture. KLF-4 expression was not significant. ** = *p* < 0.01, *ns* not significant

Serial passaging was used to examine the capacity of generated spheroids to form secondary spheres. Spheroids produced from PC-3 cells still exhibited the ability to generate spheroids after four sub-cultures, as shown by the seeding of single cells from the early passage of spheroids (Fig. 3). Thus, these data propose that spheroids preserve their self-renewal capacity over several passages. Furthermore, RT-qPCR analysis validated a definitive up-regulation in examined stem cell-related genes SOX2, OCT4, NANOG, and C-Myc, in PC-3 spheroids compared to the 2D monolayers. PC-3 spheroids displayed the significant up-regulation of SOX2, C-MYC, NANOG, and OCT4 (*p* < 0.01; Fig. 4), except KLF-4.

**Fig. 5:**
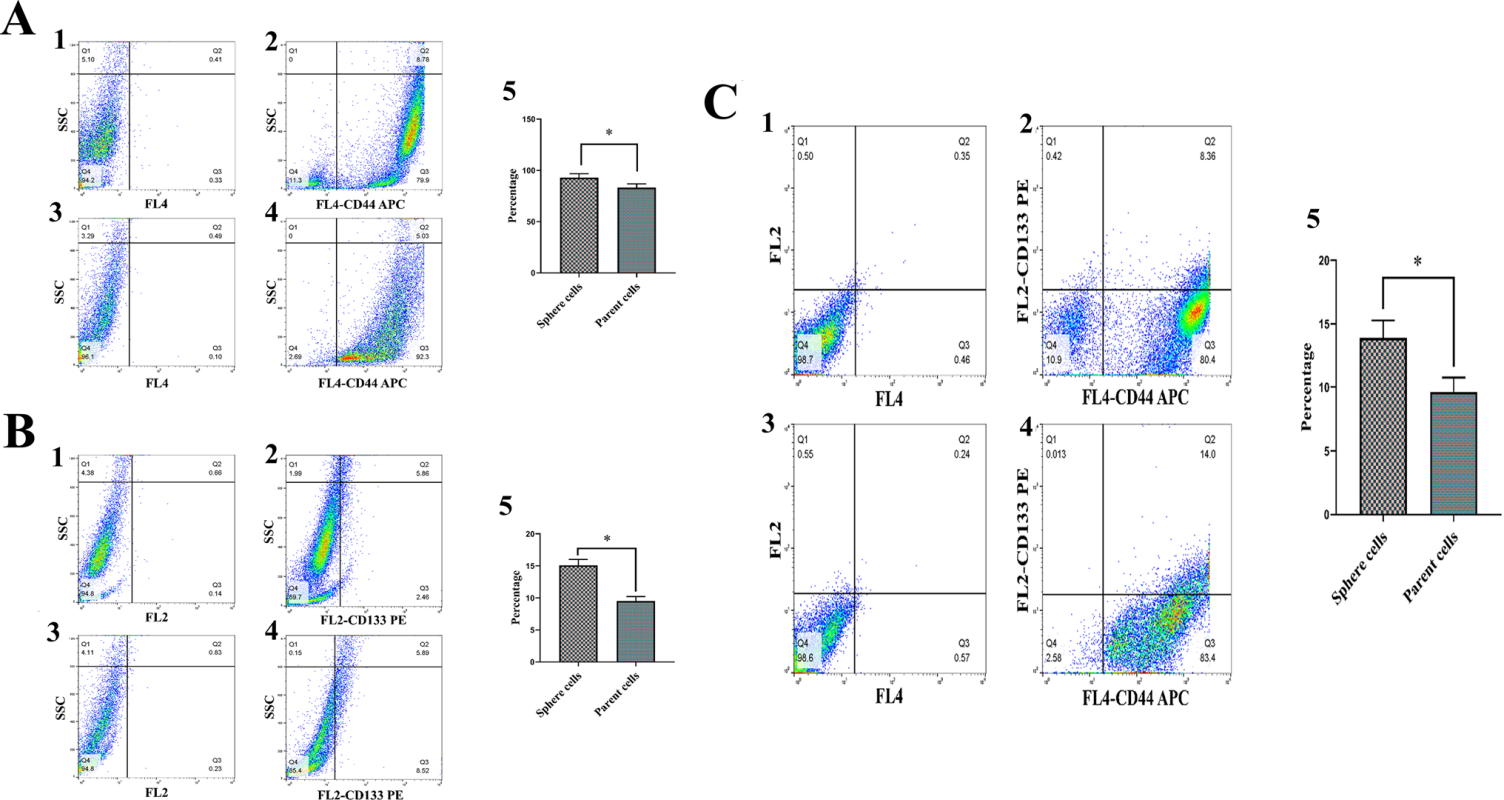
Flow cytometry analysis of cancer stem cell markers CD44 and CD133 expression as single positive (CD44^+^ or CD133^+^) and double positive (CD44^+^CD133^+^) in PC-3 spheroids compared to their parental cells. Representative flow cytometry plots of PC-3 spheroids show higher expression of CD44, and CD133 markers compared to their parental cells. Dot plots show the expression of each marker in one representative experiment. **A** CD44^+^ parental cells vs CD44^+^ spheres. **A1** Negative control (unstained cells) of parental cells. **A2** CD44 staining of parental cells. **A3** Negative control (unstained cells) of spheres. **A4** CD44 staining of spheres. **A5** Percentage of CD44^+^ spheres was significantly higher than CD44^+^ parental cells (* = P < 0.05). **B** CD133^+^ parental cells vs CD133^+^ spheres. **B1** Negative control (unstained cells) of parental cells. **B2** CD133 staining of parental cells. **B3** Negative control (unstained cells) of spheres. **B4** CD133 staining of spheres. **B5** Percentage of CD133^+^ spheres was significantly higher than CD133^+^ parental cells (* = P < 0.05). **C** CD44^+^CD133^+^ parental cells vs CD44^+^CD133^+^ spheres. **C1** Negative control (unstained cells) of parental cells. **C2** CD44 and CD133 staining of parental cells. **C3** Negative control (unstained cells) of spheres. **C4** CD44 and CD133 staining of spheres. **C5** Percentage of CD44^+^CD133^+^ spheres was significantly higher than CD44^+^CD133^+^ parental cells (* = *p* < 0.05)

The expression of CSC surface markers CD44 and CD133 was examined to further support these findings. Similar to our findings from the stemness gene expression investigation, the results of the flow cytometry analysis showed that the expression of the CSC markers CD44 and CD133 was significantly higher in spheroid cultures than in differentiated 2D cultures (*p* < 0.05; Fig. 5A, B, C).

### PLAC-1 is up-regulated in CSC-enriched spheroids compared to parental cells

**Fig. 6:**
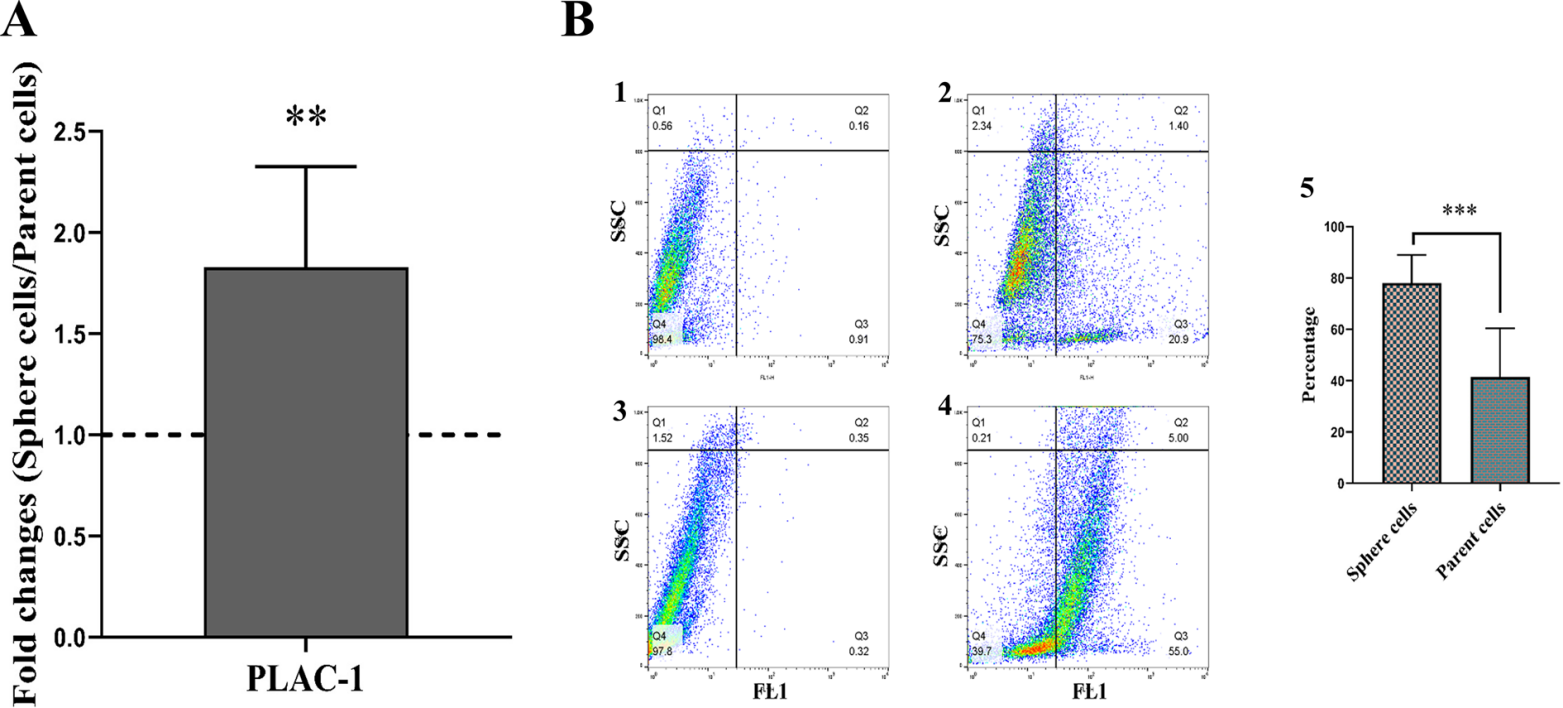
Increased expression of PLAC-1 in PC-3-derived spheroids compared to adherent counterparts. **A** Quantitative real-time PCR analysis of PC-3 spheroids revealed the higher expression of PLAC-1 compared to PC-3 parental cells (** = P < 0.01). Data are presented as mean ± SD from three independent experiments. The fold change of genes in cell spheroids was investigated after 14 days of culture. **B** Flow cytometry analysis of PLAC-1 expression in PC-3 spheroids compared to their parental cells. Representative flow cytometry plots of PC-3 spheroids show higher expression of PLAC-1 compared to their parental cells. Dot plots show the expression of PLAC-1 in one representative experiment. **B1** Negative control (unstained cells) of parental cells. **B2** PLAC-1 staining of parental cells. **B3** Negative control (unstained cells) of spheres. **B4** PLAC-1 staining of spheres. **B5** Percentage of PLAC-1^+^ spheres was significantly higher than PLAC-1^+^ parental cells (*** = *p* < 0.001)

The expression of the PLAC-1 molecule in PCSCs and parental cells of PC-3 prostate cancer cell line was investigated at the gene level. The analysis of RT-qPCR results showed that the expression of PLAC-1 molecule was significantly increased in the spheroid colonies derived from the PC-3 cell line compared to the parental cells (*p* < 0.01; Fig. 6A).

At the protein level, PLAC-1 expression in enriched CSCs and parental cells of the prostate cancer cell line PC-3 was examined. According to the analysis of the flow cytometry findings, in the spheroid colonies compared to the parental cells, PLAC-1 expression significantly increased (*p* < 0.001; Fig. 6B).

## Discussion

Our increasing understanding of how CSCs contribute to tumor progression, particularly PC, has been instrumental in designing immunotherapeutic approaches that target cancer antigens. The preclinical and clinical successes of these approaches stem from decades of research focused on understanding CSCs biology and mechanisms governing tumor progression. In this study, we show for the first time that PLAC-1 has a fundamental expression in PCSCs at the levels of gene and protein. Indeed, we demonstrate that the expression of PLAC-1 is higher in the PCSCs-enriched population compared to their parental PC cells in the human prostatic adenocarcinoma PC-3 cell line.

It is well established that PLAC-1 is activated in many human solid tumors [10, 11, 20, 39, 49], contributing to malignant cell processes such as proliferation, motility, migration, invasion, and metastasis [11]. A previous study demonstrated high expression of PLAC-1 in high-grade prostate adenocarcinoma. In other words, PLAC-1 expression is up-regulated in accordance with increasing grade and Gleason score in tissues obtained from patients with PC [40]. Given the role of PCSCs in PC progression and malignancy [54], it was hypothesized that the expression of PLAC-1 might be up-regulated on PCSCs, and our findings accentuate this hypothesis, which has not previously been described.

The role of the PLAC-1 in the context of trophoblast invasion has been a subject of considerable interest in recent research. Studies utilizing cell invasion and migration assays have demonstrated that PLAC-1 plays a role in the process of primary extravillous trophoblasts invading the maternal decidua. This was observed in the HTR8/SVneo trophoblast cell line, providing compelling evidence of PLAC-1’s involvement in this critical biological process [55]. Moreover, a comparative study using a cDNA subtraction library between invasive and non-invasive murine trophoblasts identified PLAC-1 as a key gene associated with trophoblast invasion [56]. Intriguingly, these findings opened up new avenues of research, prompting scientists to investigate if PLAC-1 could play a similar role in the progression of cancer [10]. The research conducted by Koslowski and colleagues has shed light on the pivotal role of the PLAC-1 gene in the motility, migration, and invasion of breast cancer cells, specifically MCF-7 and BT-549. By employing siRNA silencing of PLAC-1, they were able to demonstrate a profound dependency of these cellular processes on PLAC-1 expression. Remarkably, the silencing of PLAC-1 induced a G1-S cell cycle arrest, leading to an almost complete halt in cell proliferation. Further confirmation of these findings was obtained by inhibiting the proliferation of MCF-7 and BT-549 cells using a blocking rabbit anti-PLAC-1 antibody, which targets amino acids 117–127 [11]. Yang and colleagues demonstrated that the suppression of PLAC-1 in non-small cell lung cancer cells hindered cell growth, triggered apoptosis, and partially reduced the invasive capability through the control of EMT-associated proteins like E-cadherin and vimentin [27]. Yongfei and colleagues conducted further research on the role of PLAC-1 in breast cancer cell invasion and metastasis. They discovered that PLAC-1 interacts with Furin, which results in the degradation of Notch1 and the production of Notch1 intracellular domain (NICD) fragments. These fragments have the ability to inhibit the activity of PTEN, a recognized tumor suppressor protein. The inhibition of PTEN fosters cell invasion and metastasis [23]. This highlights the significant role of PLAC-1 in promoting the progression of breast cancer. In addition, the silencing of PLAC-1 through siRNA or the blocking of PLAC-1 using an anti-PLAC-1 antibody led to a decrease in the levels of phosphorylated protein kinase B, also referred to as AKT [11, 27, 29]. This implies that the activation of the AKT kinase plays a role in functioning the downstream effects of PLAC-1. All in all, the expression of PLAC-1 is notably controlled by signaling pathways that are dependent on mitogen-activated protein kinase (MAPK) and phosphoinositide 3-kinase (PI3-K) [10]. Moreover, MAPK signaling pathway is responsible for CSCs development and maintenance [57]. Thus, this may explain the dysregulation of PLAC-1 on PCSCs. However, further research is needed to elucidate the potential reasons for the increased expression of PLAC-1 in PCSCs.

Two studies showed that the PLAC-1 molecule increases the proliferation, migration, and invasion of laryngeal and breast cancer cells through the Furin/NICD/PTEN pathway [23, 28]. Another study revealed that the PLAC-1 molecule via FGFR2IIIb activates AKT phosphorylation in cancer cell lines, which in turn increases cancer cell proliferation [24]. Liu et al. discovered that the PLAC-1 molecule increases the proliferation of gastric cancer cells through the AKT/GSK-3β/cyclin D1-directed pathway [39]. Another study reported that the PLAC-1 molecule increases metastatic potential and is associated with the PI3K/AKT/NF-kB pathway in colon cancer [33]. A study based on bioinformatics data analysis concluded that the PLAC-1 molecule is a potential molecular target for head and neck cancer [58]. A study by Mahmoudi et al. in 2020 showed the high expression of the PLAC-1 molecule in the tissues of melanoma patients. This study reported that PLAC-1-based immunotherapy exerted cytotoxicity in melanoma cells [19]. Similarly, another study in 2017 showed that immunotherapy based on targeting the PLAC-1 molecule causes the destruction of prostate cancer cells [41]. According to the aforementioned evidence and findings of current study, CSCs immunotherapy targeting PLAC-1 might be considered as one of the future strategy in the field of tumor targeted therapy.

A project by NCI prioritized ideal cancer antigens based on several criteria, as mentioned in the review of the literature. One of these criteria is the expression of cancer antigens in stem cells. In this prioritization, PLAC-1 was ranked 47th among 75 candidates [52]. Notably extensive studies had not been conducted regarding the association between PLAC-1 and oncogenesis until the NCI project. Given the findings of the current study indicating the expression of PLAC-1 on PCSCs, we suggest the future studies focus on the expression of PLAC-1 on CSCs in a spectrum of solid tumors. Current study findings may support promoting the PLAC-1 position hierarchically in this prioritization.

Recently, numerous preclinical evidence has revealed that CSCs can be introduced as potential therapeutic targets [59–61]. These cells could be targeted by immunotherapeutic approaches, probably leading to complete regression of tumors and dramatic anti-tumor responses. As a specific example, EpCAM-expressing PCSCs were eradicated by EpCAM-specific chimeric antigen receptor (CAR) T cells in PC3M and PC-3 tumor models [62]. Accordingly, the development of immunotherapeutic approaches targeting CSCs entails the identification of novel molecular targets, which are specifically expressed by tumors and not by normal cells. Thus, an initial objective of the current study was to identify PLAC-1 as a potential target on PCSCs. Previous results firmly indicate that no detectable expression of PLAC-1 could be discovered in most normal tissues, including the prostate [11, 40, 63], making PLAC-1 an attractive molecular target. Nejadmoghaddam et al. [41] demonstrated that PLAC-1 is a target for immunotherapy based on antibody-SN38 drug conjugate for treating PCs. They found that anti-PLAC-1 antibody conjugated with SN38 induced apoptosis in human primary PC cells and PC cell lines such as PC-3, LNCaP, and DU145 [41]. The findings of this study might be an important cornerstone for developing a novel immunotherapeutic modality based on PLAC-1 for targeting CSCs, particularly PCSCs. Future studies on the current topic are therefore recommended.

Cancer stemness features account for the relative aggressiveness of tumors. A potential problem regarding the identification of these cells is that CSC markers may not be specific on their own, and may need to be used along with other surface markers [64]. To solve this problem, we used co-immunostaining of CD44 and CD133 molecules with flow cytometry in addition to the gene expression of putative CSC markers, including SOX2, OCT4, NANOG, C-MYC, and KLF-4. The findings of the current study corroborated the expression profiles of stem cell-related genes, which are evidenced by enriching the CD44 and CD133-positive populations of CSCs in prostaspheres.

In current study, for enriching PCSCs derived from the PC-3 cell line, the method of forming free-floating spheroid colonies in serum-free medium in a non-adherent substrate (3D cell culture) was used. For studying the properties of CSCs in vitro, 3D cell culture methods based on SFA have recently gained prominent popularity [65, 66]. SFA has been extensively used for enriching CSCs in a wide variety of tumors including prostate [67], brain [68, 69], breast [70], lung [71], head and neck [72], and melanoma [73]. This type of cell culture is a decent model for discovering the characteristics of CSCs [74]. Among these characteristics, it can be mentioned ability of self-renewal, the high expression of genes related to stemness/multipotency and surface molecules [9], which in this study, all these features were proved through serial passages, the evaluation of SOX2, OCT4, Nanog, C-Myc and KLF-4 expression, and the assessment of CD44 and CD133 expression.

The formation of spheroid colonies during serial passages is used as a suitable method for long-term proliferation of cells with self-renewal capacity and mimicking tumor heterogeneity conditions [53, 65]. In this study, spheroid colonies derived from the PC-3 cell line were serially passaged in the culture medium until 4 passages, and the cancer cells maintained their ability to proliferation. Therefore, this finding supports the efficiency of serum-free and non-adherent culture medium for enriching PCSCs. Additionally, in this study, PCSC colonies derived from the PC-3 cell line formed large colonies with irregular edges and loose cell organizations. This morphology of colonies was consistent with other studies [75–78].

SOX2 is known as an undifferentiated cell marker. Studies have shown that SOX2 plays a role in maintaining CSCs and tumor malignancy [79, 80]. OCT4 is a key regulatory gene that maintains the pluripotency and self-renewal properties of embryonic stem cells. Although evidence suggests that OCT4 acts as an oncogene in several types of cancer [81, 82]. Nanog is a key regulator of stem cell development and cell reprogramming, and its expression is directly related to malignancy in many cancers [83]. The C-Myc molecule is an oncogene that acts as a regulator of the cell cycle and cell death pathway in various cancers [84] and maintains PCSCs [85]. In current investigation, the expression of SOX2, OCT4, Nanog, and C-Myc in spheroids population was significantly higher than in the tumor parental population. The results of other studies agreed with the findings of this study [75, 85, 86]. Nanog protein is expressed in prostate cancer cells and is enriched in CD44^+^ and CD44^+^/CD133^+^ cells [87]. This finding is consistent with the findings of the present study.

Although KLF-4 has been shown to act as a tumor suppressor in PC [88, 89], its function during initiation and progression of the PC has not been fully elucidated. In this study, KLF-4 gene expression was increased in the group of PCSCs compared to the parental tumor cells. However, this increment was not statistically significant. A study has reported that KLF-4 is highly expressed in malignant prostate cancer at the gene and protein levels. This increase in expression in the PC-3 cell line was higher than most of the prostate cancer cell lines used in the same study [90]. Also, KLF-4 expression was higher in prostate cancer than in benign prostatic hyperplasia [90]. A study has shown that KLF-4 inhibits the malignant progression of prostate cancer and is a good predictor of prognosis in this type of cancer [91].

As an adhesion receptor for hyaluronic acid, the CD44 molecule binds to the extracellular matrix and plays a paramount role in matrix adhesion. It also increases cell accumulation and is known as a marker for CSCs [92, 93]. CD133 molecule is an adhesion molecule, and CD133^+^ human PC cells can initiate cancer [94]. Additionally, CD133^+^ cells can self-renew and form spheroid colonies composed of CSCs [95]. Since cell adhesion proteins participate in intercellular junctions, it can be assumed that specific and tight cell junctions in spheroids derived from PC-3 cell line may be related to the high expression of CD44 and CD133 molecules in these colonies. These junctions between cells may be responsible for maintaining the phenotype of PCSCs.

The current study was a preliminary report that might provide a fundamental framework for future studies. Thus, several significant limitations need to be considered. First, in this study, only one type of cell line named PC-3 was used, which is an androgen-independent cell line. Thus, it is suggested to use other cell lines such as DU145, LNCaP (an androgen-dependent cell line), and VCaP in future studies. Also, using control cell lines like RWPE-1 can provide further data demonstrating the difference between the expressions of PLAC-1 in different contexts. Second, the study is limited by the lack of information on various aspects and functional assays for tumorigenesis, oncogenic transformation, and cancer stemness in an animal model for PC. Lastly, methods of gene knockdown/out, such as the use of siRNA or RNA interference, could also serve to further confirm the role of PLAC-1 in prostate cancer progression.

## Conclusion and future directions

In conclusion, we have demonstrated for the first time that PLAC-1 is up-regulated in PC-3 cell line derived-PCSCs. Based on the data presented in this study, it can be hypothesized that PLAC-1 may serve as a potential target for therapies aimed at prostate cancer. However, additional studies are required to elucidate the underlying mechanisms of PLAC-1 in PCSCs, gain a more comprehensive understanding of the associated signaling pathways, and develop a deeper appreciation of the PLAC-1-specific immune response in CSCs. For future studies, it is recommended to investigate the expression of the PLAC-1 molecule in CSCs derived from other cancer cell lines and samples from cancer patients. Also, functional studies and immunotherapy approaches based on targeting the PLAC-1 molecule on PCSCs may be performed in vitro and in vivo. Additionally, the effects of targeting PLAC-1 molecule on tumor growth and spread may be evaluated. Future research findings may pave the way for PLAC-1 to be recognized as a potential treatment target during both preclinical and clinical phases.

## Data Availability

The datasets generated during and/or analysed during the current study are available from the corresponding author on reasonable request.

## Abbreviations

SFA: Sphere-formation assay
CSC: Cancer stem cell
PCSC: Prostate cancer stem cell
PLAC-1: Placenta-specific protein 1
PC: Prostate cancer
PBS: Phosphate buffered saline
EGF: Epidermal growth factor
bFGF: Basic fibroblast growth factor
